# Sex-Specific Autonomic Responses to Acute Resistance Exercise

**DOI:** 10.3390/medicina57040307

**Published:** 2021-03-24

**Authors:** Stacie M. Humm, Emily K. Erb, Emily C. Tagesen, J. Derek Kingsley

**Affiliations:** Cardiovascular Dynamics Laboratory, Exercise Science/Physiology, Kent State University, Kent, OH 44242, USA; shumm2@kent.edu (S.M.H.); Eerb4@kent.edu (E.K.E.); Etagesen@kent.edu (E.C.T.)

**Keywords:** vagal modulation, sympathovagal balance, autonomic modulation, maximal strength

## Abstract

*Background and Objectives:* Acute resistance exercise (RE) reduces vagal modulation and increases sympathovagal balance, which increases the risk for arrythmias. Few studies have examined sex differences in autonomic modulation after acute RE. The purpose of this investigation was to examine sex-specific responses to acute RE on autonomic modulation. *Materials and Methods:* Twenty-one resistance-trained individuals (men n = 11, women n = 10) between the ages of 19 and 25 y were analyzed for autonomic modulation in response to acute RE and a control (CON). Measures of autonomic modulation were collected at rest, 15 (R15), and 30 (R30) min following both conditions. Heart rate (HR), log transformed root mean square of successive differences (lnRMSSD), total power (lnTP), low-frequency power (lnLF), high-frequency power (lnHF), sample entropy (SampEn), and Lempel-Ziv entropy (LZEn) were measured at all time points. A three-way repeated analysis of variance (ANOVA) was used to analyze sex (men, women) across condition (RE, CON) and time (Rest, R15, R30). *Results:* The results are similar for all heart rate variability (HRV) variables at rest for both conditions (RE, CON). SampEn was significantly higher in men compared to women at rest for both conditions (*p* = 0.03), with no differences in LZEn (*p* > 0.05). There were no significant (*p* > 0.05) three-way interactions on any variables. Condition by time interactions demonstrated that both sexes increase in HR (*p* = 0.0001) and lnLF/HF ratio (*p* = 0.001), but decreases in lnRMSSD (*p* = 0.0001), lnTP (*p* < 0.0001), lnLF (*p* < 0.0001), lnHF (*p* = 0.0001), and LZEn (*p* = 0.009) at R15 and R30 compared to rest following acute RE and were different from CON. Condition by time interaction (*p* = 0.017) demonstrated that SampEn was attenuated at R15 compared to rest, and the CON, but not R30 following acute RE. *Conclusion:* Although SampEn is more complex at rest in men compared to women, autonomic modulation responses between sexes following acute RE appear to be similar.

## 1. Introduction

Health benefits of resistance exercise (RE) are well-known, and its use is recommended by professional organizations in young, healthy individuals as a means to increase maximal strength and muscle hypertrophy [1]. However, it has been suggested that an acute bout of resistance exercise produces significant reductions in vagal modulation as measured by heart rate variability (HRV) [2,3,4,5] and heart rate complexity (HRC) [2,3,4,5,6]. Heart rate variability refers to the linear variations in the R-R interval [7]. However, within these HRV signal spectra, not all of the data are harmonic, as randomness or irregularity inherently exists within the system [8]. Heart rate complexity focuses on the non-linear, irregularity of the R-R interval and thus may be used to detect changes in autonomic modulation that were lost via HRV [8].

Reductions in both HRV and HRC are mediated primarily by vagal tone [7,8]. Decreases in vagal modulation are associated with the onset of paroxysmal atrial fibrillation [9] and cardiac arrhythmias. Interestingly, it has also been postulated that HRC provides a more sensitive, robust measure of autonomic modulation compared to HRV measures in the same time period [10].

Specifically, an acute bout of RE reduces linear and non-linear measures of vagal modulation as well as increases sympathovagal balance for at least 30 min [3,4,5,6]. Heffernan et al. (2006) reported that eight exercises for the whole-body using a mixture of machine and free-weight resistance exercises reduces vagal modulation and increases sympathovagal balance for at least 30 min as measured via HRV in young active men. Similarly, Kingsley et al. (2014) demonstrated that four exercises for the whole-body using weight machines in resistance-trained individuals also reduced vagal modulation as measured by HRV and HRC, and increased sympathovagal balance for at least 30 min. Collectively, these data demonstrate that vagal tone is reduced for at least 30 min, with increases in sympathovagal balance, following an acute bout of RE in active individuals. However, few studies have sought to determine if there are sex-specific autonomic responses in active individuals following acute RE [11].

While it has been demonstrated that young, healthy, sedentary women have greater vagal modulation [12] and reduced sympathovagal balance [13,14] at rest compared to men [15] these comparisons in active individuals are severely limited. Kingsley et al. (2019) and Teixeira et al. (2011) reported no significant differences at rest between sexes when the individuals were resistance-trained, or active, respectively. To our knowledge no studies have evaluated resting measures of autonomic modulation between the sexes using HRC, which again may be a more sensitive measure and may provide further insight into sex-specific autonomic modulation. It is clear that gaining more data evaluating sex differences in active individuals at rest is both pertinent and meritorious.

Data on autonomic modulation in response to acute RE between the sexes is very limited. Following a bout of free-weight RE, Kingsley et al. (2019) reported no differences between the resistance-trained sexes for vagal modulation or sympathovagal balance measured via HRV up to 30 min. In addition, we are unaware of any studies that have examined HRV and HRC in response to acute weight-machine RE between the sexes. Therefore, this study sought to examine differences between the sexes on autonomic modulation using HRV and HRC at rest, and up to 30 min during recovery from an acute bout of whole-body RE using weight-machines in active men and women. We hypothesized that there would be no differences in resting measures of autonomic modulation between the sexes as measured using HRV and HRC. We further hypothesized that an acute bout of weight-machine RE would reduce vagal modulation and increase sympathovagal balance as measured by HRV and reduce vagal modulation as measured by HRC for at least 30 min. Lastly, we hypothesized that women would have attenuated measures of autonomic modulation measured via HRC in response to the acute RE compared to men.

## 2. Materials and Methods

### 2.1. Participants

Eleven men and 10 women participated in the study. Participants were recruited by flyers placed around campus and by word of mouth. Participants were excluded if they had any cardiovascular, respiratory, renal, musculoskeletal, or metabolic diagnoses, as well as uncontrolled hypertension (SBP ≥ 140 mmHg, DBP ≥ 90 mmHg) and obesity (Body mass index ≥ 30 kg/m^2^). All participants were free from medications or supplements that may alter HR or blood pressure (BP) as assessed via a Health History Questionnaire [16]. Based on the Lipids Research Questionnaire [17] all participants were classified as moderately active. Estradiol concentrations are lowest during the first nine days of a woman’s menstrual cycle. Thus, to control for any effects of estrogen on autonomic modulation, all women were tested during the early-to-mid follicular phase (days 1–9) of their menstrual cycle based on their last menses [18]. Informed consent was signed by all participants, and all procedures utilized were approved by Kent State University and were in accordance with the Declaration of Helsinki: (IRB: 13-513; 18 December 2016).

### 2.2. Study Design

**Figure 1:**
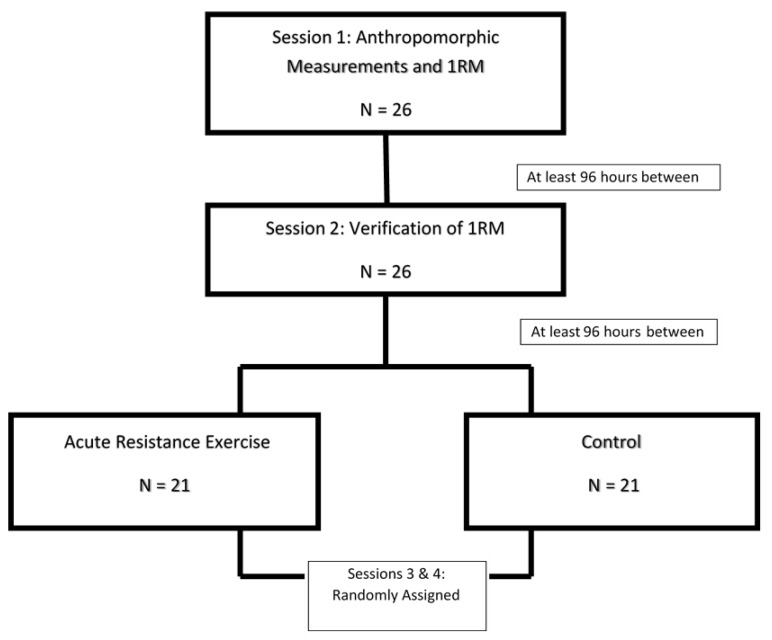
Flowchart of study design. 1RM—one-repetition maximum.

The present study utilized a randomized, cross-over design in which each participant underwent two days of maximal strength testing and verification, followed by two test days, an experimental protocol and a control (CON) (Figure 1). On those test days the participants arrived at the laboratory after a 3 h fast, having abstained from caffeine and alcohol ingestion as well as strenuous exercise for at least 24 h. All participants were tested between 6 a.m. and noon to control for diurnal variation. The second visit was ±1 h of the first visit.

### 2.3. Anthropometrics

Height and weight were measured using a calibrated balance beam scale (Detecto 448; Cardinal Scale Manufacturing, Web City, MO, USA) to the nearest mm and lb., respectively. Weight was converted into kg for quantification of BMI (kg/m^2^).

### 2.4. Maximal Strength

Maximal strength was assessed using the one-repetition maximum (1RM) as outlined by the National Strength and Conditioning Association (NSCA) [19] using Cybex (Medway, MA, USA) sectorized chest press, leg press, lat pulldown, leg curl, and leg extension. In short, each participant underwent a warmup on a cycle ergometer at 50W for five min prior to performing 6–8 repetitions at 50–70% of the body weight. Weight on the machines was increased by 5% for upper-, and 10% for lower-body until a weight could be moved only one time. A total of five attempts was allowed, with 3 min of rest between attempts and exercises. Verification of the 1RM was accomplished at least 96 h later, and no more than 1 week.

### 2.5. Autonomic Modulation

After a minimum of 96 h from the 1RM verification participants returned to the laboratory for their first condition. After a 10 min rest period in the supine position, autonomic modulation was measured for five min using a modified CM5 3-lead electrocardiogram (ECG) (ADInstruments, Powerlab, Colorado Springs, CO, USA) sampling at 1000 Hz. Immediately after resistance exercise, participants returned to the supine position. At both 15 min post exercise (R15) and 30 min post exercise (R30), autonomic modulation was quantified. Autonomic modulation was assessed with the use of HRV and HRC using an ECG configuration (WinCPRS, Absolute Aliens, Turku, Finland) at a rate of 1000 Hz. Before importing to WinCPRS, the ECGs were inspected for artifact and ectopic beats. Ectopic beats were then removed and interpolated. A metronome was set to regulate the participant’s breathing at 12 breaths/min while obtaining the ECG.

Heart rate variability is an analysis of the variations in the intervals between successive R waves on the ECG and is an estimation of the global activity of the autonomic nervous system [7]. For the frequency domain, total power (TP) of HRV was used as an index for overall autonomic modulation [7]. Low-frequency power (LF) was between 0.04–0.15 Hz and was mediated by both sympathetic and parasympathetic branches of the autonomic nervous system [7]. High-frequency power (HF) was between 0.15 and 0.4 Hz and was indicative of parasympathetic modulation and was used as a measurement of vagal modulation [7]. For the time domain, root mean square of successive differences of normal to normal intervals (RMSSD) and the number of normal to normal intervals which differed by >50 ms from adjacent intervals divided by the total number of all normal to normal intervals were used as a measure of vagal modulation [7]. The LF/HF ratio was used to measure sympathovagal balance [7].

Sample entropy (SampEn) and Lempel-Ziv entropy (LZEn) were used to evaluate the R waves following removal of the linear trend [8]. Specifically, it has been shown that the more predictive the ECG signal, the closer the value of SampEn is to 0, while a more chaotic signal is closer to 2. Thus, the fewer matches or sequences there are, the more chaotic the signal, which is indicative of a vagal tone [8].

Lempel-Ziv entropy is calculated using the same data points on the Poincare plot as SampEn [8]. This variable, using Kolmogorov estimates, evaluates the differing and repeating patterns, from long to short, to produce binary code in which ‘1’ is value above the mean, or ‘0’ when it is below the mean. From this binary code, LZEn can be obtained and its interpretation is similar to that of SampEn in that lower values are fixed, while higher values are more chaotic [8].

### 2.6. Acute Resistance Exercise

The present study had participants complete three sets of 10 repetitions at 70% 1RM for the five resistance exercises. Rest breaks between sets were two min, with three min of rest between exercises. If participants were unable to complete the 10 repetitions, then the weight was reduced on the subsequent set.

### 2.7. Statistics

Sex differences for descriptive data and baseline characteristics were evaluated with independent samples *t*-tests. A 2 × 2 × 3 three-way analysis of variance (ANOVA) was used to examine differences between men and women during two randomized conditions (acute resistance exercise, CON) with a repeated measure of time (Rest, R15, R30). If the ANOVA was significant post hoc testing utilized t-tests with a Fisher LSD correction factor. All analyses were done using SPSS v.26 (Armonk, NY, USA). Alpha was set apriori at *p* ≤ 0.05. Partial eta squared (η^2^) was reported as a measurement of effect size for the repeated measures ANOVA and 95% confidence intervals (CI) were used for the independent and paired samples t-tests. All data are reported as mean ± SD. Data were evaluated for normality using a Shapiro–Wilk analysis. The data that were not normally distributed included RMSSD, TP, LF, HF, and the LF/HF ratio. Accordingly, they were log transformed (ln). If sphericity was not met, then a Greenhouse–Geiser adjustment was utilized. Based on previous data reported on the effects of resistance exercise on vagal modulation using HF power [3] with an alpha of 0.05, and a power of 80%, we determined a Cohen’s d of 1.22. This required a minimum of 10 participants per sex. Thirteen participants per sex were recruited; however, five subjects (2-men, 3-women) were unable to complete the protocol, leaving us with n = 21.

## 3. Results

Participant characteristics are presented in Table 1. Men (n = 11) had significantly (*p* < 0.05) greater height and weight than women (n = 10), and greater 1RM on all resistance exercises (Table 2). They were similar (*p* > 0.05) for age and BMI.

**Figure 2:**
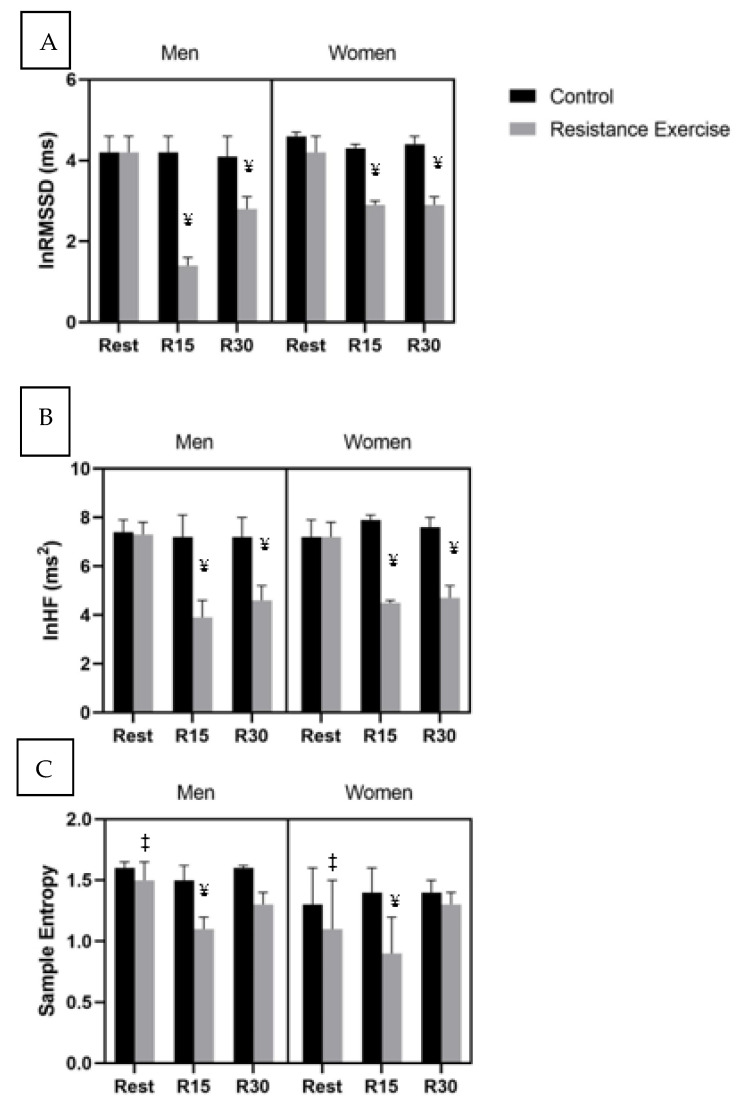
Alterations in vagal modulation (mean ± 95% confidence intervals) measured via (**A**) ln root means square of successive differences (lnRMSSD), (**B**), ln high-frequency power (lnHF), and (**C**) sample entropy at rest, as well as 15 (R15), and 30 min following a control and an acute bout of resistance exercise in resistance-trained men (n = 11) and women (n = 10). Values are mean ± SD. ¥ significantly different from rest (*p* = 0.0001), ‡ significantly different from men (*p* = 0.03).

Heart rate and autonomic data are presented in Table 3 and vagal modulation data are presented in Figure 2. The sexes were similar on all HRV variables at rest for both conditions (*p* > 0.05). SampEn was augmented in the men compared to the women at rest for both conditions (*p* = 0.03) and with no differences in LZEn. There were no significant (*p* > 0.05) three-way interactions on HR or measures of autonomic modulation as measured via HRV or HRC. As there were no significant main effects of sex, the sexes were combined for the presentation of the analysis. There was a significant condition–time interaction for HR (F_2,38_ = 130.50, *p* = 0.0001, η^2^ = 0.89), such that it was increased following the resistance exercise condition at R15 and R30 compared to Rest, and R15 was higher compared to R30 with no change across time during the CON. In regard to HRV variables there were significant condition x time interactions for lnRMSSD (F_2,38_ = 51.36 *p* = 0.0001, η^2^ = 0.73), lnTP (F_2,21_ = 47.48, *p* < 0.0001, η^2^ = 0.94), lnLF (F_2,38_ = 20.11, *p* < 0.0001, η^2^ = 0.51), lnHF (F_2,38_ = 93.17, *p* = 0.0001, η^2^ = 0.83), and lnLF/HF (F_2,38_ = 8.20, *p* = 0.001, η^2^ = 0.30). Natural log RMSSD, lnTP, lnLF, and lnHF were significantly lower at R15 compared to rest and CON following acute RE compared to rest and CON. Recovery at thirty minutes (R30) after acute RE was also different than rest for lnRMSSD, lnTP, lnLF, and lnHF and compared to the CON. There was also a significant increase in lnTP and lnLF from R15 to R30, such that although they increased, they did not statistically return to rest. Natural log LF/HF was significantly greater at R15 and R30 following acute RE compared to rest and CON. The HRC data demonstrated significant condition by time interactions for SampEn (F_2,38_ = 4.57, *p* = 0.017, η^2^ = 0.19) and LZEn (F_2,38_ = 6.38, *p* = 0.009, η^2^ = 0.44). Following acute RE, SampEn was significantly reduced at R15 compared to rest and R30, with no changes following CON. LZEn was reduced at R15 and R30 compared to rest following the acute RE but not the CON. In addition, LZEn was statistically increased at R30 compared to R15.

## 4. Discussion

The major findings of this study are that (a) there no sex-specific differences for resting values of autonomic modulation when using HRV, but HRC using SampEn demonstrated that men were more complex compared to women at rest, (b) there were significant reductions in vagal tone that were similar between the sexes following the acute bout of resistance exercise, (c) significant increases in sympathovagal balance were also similar between sexes after the acute bout of resistance exercise. Collectively, these data demonstrate that even though SampEn was different at rest between the sexes, suggesting augmented vagal tone in men, autonomic modulation responded similarly between the sexes to the acute bout of resistance exercise.

There is a dearth of knowledge on the differences in HRV at rest in young, healthy active men and women [5,11,20]. In agreement with our hypothesis our data suggest that in active individuals there is no difference in resting autonomic modulation between the sexes assessed via HRV. Voss et al. [12] reported that at rest young women had greater HF, as well as a lower LF/HF ratio, compared to young men. However, the work by Voss et al. (2015) did not include the activity level of their participants, whereas in the present study our participants were characterized as being active. Mendonca et al. (2010) also demonstrated that healthy sedentary women in the follicular phase had a greater HF and lower LF at rest compared to healthy sedentary men. Similar to the present study, Kingsley et al. (2019) also reported no differences at rest between sexes using resistance-trained participants. Teixeira et al. (2011) also reported no differences in measures of autonomic modulation in active men and women, matching the findings of the present study. Unlike the present student, a previous investigation demonstrated young women demonstrated a lower LF/HF ratio than young men at rest [21]. The present investigation suggests that there are no sex-specific differences in resting measures of HRV between active men or women which is supported by previous work but presents a very different perspective compared to those studies using sedentary research participants.

The present sought to compare sex differences on vagal modulation and sympathovagal balance during recovery from resistance exercise measured via HRV in active men and women. Our study is supported by previous work by Kingsley et al. (2019) that indicated resistance-trained women and men had similar reductions in lnRMSSD and lnHF, with concomitant increases in the lnLF/HF ratio, during recovery from free-weight resistance exercise. In this particular study, resistance-trained men and women underwent three sets of 10 repetitions at 75% 1RM on the bench press, squat, and deadlift with autonomic measurements taken at 15–20 m and 25–30 m, similar data collection times as the present study. While it is difficult to directly compare the results of the present study to Kingsley et al. (2019), as free weights and weight machines are two different stimuli, the data closely correspond for the men but not for the women. In men, the differences at R15 and R30 in the present study differed from Kingsley et al. [5] for lnRMSSD by 26.3% and 14.1%, respectively. For the women, the differences compared to the present study were 64% at R15 and a difference of 77.8% at R30. Similarly, for lnHF, the differences at R15 and R30 between the two studies were 24.5% and 10.1% for the men, respectively, while the women’s differences were 31.2% and 73.5%. Collectively, these data demonstrate that an acute bout of RE reduces vagal tone, be it weight machines or free weights. It is clear that more data are needed to investigate not only sex differences in vagal responses to acute resistance exercise but also modalities.

Additional work by Kingsley et al. (2014) also demonstrated alterations in autonomic modulation in response to weight machines in resistance-trained individuals (nine women, eight men) up to 30 min. In that particular study, there were no alterations in lnTP, unlike the present study, which showed a significant decrease in lnTP after the acute RE that did recover from 15 to 30 min, but not completely to rest. Interestingly, the findings of the present study regarding lnTP do correspond with those from Heffernan et al. [6], which further highlights how more data are needed. With regard to the reductions in lnRMSSD in Kingsley et al. (2014), these were more closely matched with those of the present study in that from 25 to 30 m following the resistance exercise there were reductions of 52.4% and 33.3% for Kingsley et al. (2014) and the present study, respectively. For lnHF, Kingsley et al. (2014) reported a 19% decrease, while the present study demonstrated a 37.0% decrease. The differences in lnHF between these two studies may be driven by differences in the number of exercises (load), exercise order, as well as the research participants utilized. The present study utilized five resistance exercises compared to four in Kingsley et al. (2014). In addition, the last resistance exercise in the present study was the leg extension, whereas Kingsley et al. (2014) finished with the chest press, which may have differing responses [3], but this is inconclusive [22]. Kingsley et al. (2014) demonstrated reductions lnHF following acute upper- and lower-body RE. However, the larger decrease in lnHF was associated with the acute lower-body resistance exercise condition, which supports the findings of the present study. Future research may benefit from exploring the responses between those that are active versus those that are specifically resistance trained.

While the current study did not note any sex differences in LF/HF ratio, previous work by Mendonca et al. [14] demonstrated that women experienced a greater change in LF/HF between rest and recovery after supramaximal exercise compared to the responses of men. For lnLF/HF, men had differences of 34.2% and 60.0% at R15 and R30, while the women had differences of 32.4% and 51.3% at the same periods. Sympathovagal balance was augmented in Kingsley et al. (2014) and in the present study. Collectively, these data demonstrate that acute RE increases sympathovagal balance for at least 30 min, regardless of sex or modality.

Despite the difference in study protocols and perhaps load, the results suggest that there are no sex differences in HRV following an acute bout of resistance exercise in active individuals. In agreement with our hypothesis, both sexes had similar reductions in vagal modulation and concomitant increases in sympathovagal balance quantified via HRV. Previous work reported that sympathetic drive is proportional to the active muscle recruitment [23]. Both studies, Kingsley et al. [5] and the present study, demonstrated significant decreases in vagal modulation with increases in sympathovagal balance that were similar between the sexes. Based on these data, vagal modulation is reduced, while sympathovagal modulation is increased, following an acute bout of resistance exercise in both sexes regardless of modality, as long as intensity is similar.

Despite there being no difference between the sexes with HRC in response to acute RE, both SampEn and LZEn were reduced at 15 min following acute RE compared to Rest, but only LZEn remained significantly reduced at 30 min. In addition, LZEn did recover from 15 to 30 min, but it was not complete such that it did not return to Rest. This was contrary to our hypothesis and is not supported by previous work. Kingsley et al. (2014) reported reductions in SampEn, while Kingsley et al. (2019) reported reductions in SampEn and LZEn, 30 min following acute RE in resistance-trained individuals. Heffernan et al. [10] demonstrated that following 15 total sets of 10 repetitions at 75%1RM on the leg press and leg extension SampEn was significantly reduced in active men. On the other hand, previous work has suggested no change in SampEn regardless of set configuration if total volume is equated in resistance-trained men and women [24]. Despite the differences in protocols to previous work, this is the first study, to our knowledge, that demonstrated a complete recovery in SampEn 30 min after acute RE, such that is was no longer significantly different compared to rest. The recovery in SampEn was not matched by the recovery in LZEn as previously published. This recovery may further support the notion that HRC, specifically SampEn, may provide a more robust, sensitive measure of autonomic modulation compared to HRV. Regardless, the recovery of SampEn by 30 min is both surprising and novel. It is plausible that resistance exercise machines may provide a faster recovery of autonomic modulation compared to free-weights when quantified using SampEn, but that is beyond the scope of the present study.

Contrary to our hypothesis the reductions in HRC were also similar between the sexes following the acute bout of RE. Even though HRC has been postulated to be a more sensitive measure of autonomic modulation compared to HRV, there was no difference between the sexes for measures of HRC during recovery from the acute RE. This is the first study to evaluate sex-specific responses to acute RE using HRC, and one of a small group to evaluate HRC in relation to acute RE.

This study is not without limitations. Women were testing during the early to mid-follicular phase of their menstrual cycle, but biochemical analyses were not run on these measurements. Future studies should consider recruiting women during alternate phases of the menstrual cycle to examine the potential effect of estrogen fluctuation on measures of autonomic function and vagal modulation following resistance exercise to explore the data demonstrated in previous studies at rest [25,26]. The present study also used the LF/HF ratio as a measure of sympathovagal balance. Previous work has suggested that the LF/HF ratio may be a sympathovagal dominance [27]. Regardless, increases in the LF/HF ratio are mediated by increases in sympathetic modulation [7].

## 5. Conclusions

In conclusion, these data demonstrate that active men and women have similar resting measures of autonomic modulation measured via HRV, but not when quantified by SampEn. Furthermore, our data suggest similar responses between active sexes to acute RE quantified via HRV and HRC. These data highlight the need for monitoring reductions in vagal tone following resistance exercise, as there is clearly a reduction in tone which may be associated with an increased risk for arrhythmias.

Practically, these data show that both men and women may have an increased risk for arrythmias in the 30 min following acute RE mediated by decreases in vagal modulation and increases in sympathovagal balance. These data demonstrate an unfavorable cardiovascular response following an acute bout of resistance exercise using weight machines. In order to provide a healthier cardiovascular response, it may be necessary to separate out these exercises into multiple training days. Collectively, our data may contribute to the ability to prescribe resistance exercise in a safe, secure and precise way.

## Figures and Tables

**Table 1:** Descriptive characteristics of participants gathered during initial testing for both active men and women (n = 21).

	Men (n = 11)	Women (n = 10)
Height (m)	1.8 ± 0.1 *	1.6 ± 0.1
Weight (kg)	79 ± 14 *	60 ± 7
Age (years)	23 ± 3	22 ± 3
BMI (kg·m^2^)	25.3 ± 2.5	23.1 ± 1.4
Years Training (years)	3 ± 2	2 ± 2

Data are mean ± standard deviation; * significantly different than women (*p* ≤ 0.05). BMI = body mass index.

**Table 2:** One-repetition maximum (1RM) for all resistance exercises utilized for both active men and women (n = 21).

	Men (n = 11)	Women (n = 10)
Leg Press (kg)	169 ± 34 *	106 ± 17
Lat Pulldown (kg)	140 ± 23 *	79 ± 13
Chest Press (kg)	145 ± 26 *	64 ± 20
Leg Curl (kg)	88 ± 21 *	47 ± 10
Leg Extension (kg)	110 ± 73 *	72 ± 22

Data are mean ± standard deviation; * significantly different than women (*p* ≤ 0.05).

**Table 3:** Heart rate and autonomic modulation at rest and during recovery from a control and an acute bout of resistance exercise at 15 min (R15) and 30 min (R30) in active men and women (n = 21).

	Control			Acute Resistance Exercise		
	Rest	R15	R30	Rest	R15	R30
Heart rate (beats·min^−1^)						
Men	60 ± 12	59 ± 13	58 ± 12	62 ± 11	92 ± 11 ¥	86 ± 12 ¥
Women	66 ± 8	62 ± 8	62 ± 7	70 ± 7	90 ± 10 ¥	85 ± 8 ¥
95% CI	(59, 68)	(57, 66)	(57, 66)	(61, 71)	(85, 96)	(80, 90)
lnTotal Power (ms^2^)						
Men	8.2 ± 0.8	8.4 ± 1.0	8.2 ± 0.8	8.3 ± 0.9	5.9 ± 1.0 ¥µ	6.5 ± 0.8 ¥µ
Women	8.2 ± 1.1	8.5 ± 1.3	8.1 ± 1.6	8.3 ± 0.8	6.4 ± 0.7 ¥µ	6.7 ± 0.6 ¥µ
95% CI	(7.8, 8.7)	(7.8, 8.8)	(7.4, 8.6)	(7.8, 8.6)	(5.6, 6.6)	(6.2, 7.0)
lnLF (ms^2^)						
Men	6.9 ± 0.8	6.9 ± 0.7	6.5 ± 0.7	6.7 ± 0.8	4.9 ± 1.0 ¥µ	5.5 ± 0.9 ¥µ£
Women	6.1 ± 2.1	7.0 ± 0.0	6.2 ± 1.2	6.2 ± 2.4	5.3 ± 0.8 ¥µ	5.7 ± 0.5 ¥µ£
95% CI	(5.9, 7.1)	(5.8, 7.3)	(5.2, 7.3)	(6.1, 7.5)	(4.2, 5.7)	(5.2, 6.3)
lnLF/HF (ratio)						
Men	3.7 ± 2.0	3.9 ± 1.7	4.1 ± 0.8	3.5 ± 1.4	4.8 ± 1.9 ¥ƒ	5.1 ± 2.0 ¥ƒ
Women	3.4 ± 0.7	3.2 ± 1.8	3.2 ± 1.1	3.7 ± 0.6	5.0 ± 1.5 ¥ƒ	5.6 ± 1.1 ¥ƒ
95% CI	(3.3, 4.5)	(3.1, 4.7)	(2.9, 4.2)	(3.4, 4.7)	(4.4, 6.5)	(5.0, 6.3)
LZEntropy						
Men	0.8 ± 0.0	0.8 ± 0.1	0.7 ± 0.1	0.8 ± 0.0	0.5 ± 0.1 ¥	0.6 ± 0.1 £
Women	0.9 ± 0.3	0.9 ± 0.3	1.0 ± 0.3	1.0 ± 0.4	0.7 ± 0.2 ¥	0.9 ± 0.3 £
95% CI	(0.7, 0.8)	(0.7, 1.0)	(0.7, 1.1)	(0.7, 1.1)	(0.5, 0.8)	(0.5, 1.0)

lnLF = natural logarithm low-frequency power; LZEntropy = Lempel-Ziv entropy; Values are mean ± SD; ¥ significantly different from rest (*p* = 0.0001); ƒ significantly different from CON (*p* = 0.001); µ significantly different from CON (*p* = 0.0001); £ significantly different from R15 (*p* = 0.0001).

## Data Availability

The data presented in this study are available on request from the corresponding author. The data are not publicly available due to privacy/ethical concerns.
